# Analysis of risk factors for the development of bronchiolitis obliterans in children with severe *Mycoplasma pneumoniae* pneumonia

**DOI:** 10.3389/fmed.2026.1815375

**Published:** 2026-04-10

**Authors:** Jiapu Hou, Ruiyang Sun, Zewen Ding, Wanyu Jia, Peng Li, Shuqin Fu, Chunlan Song

**Affiliations:** Henan Provincial Children's Hospital, Zhengzhou, Henan, China

**Keywords:** bronchiolitis obliterans, children, *Mycoplasma pneumoniae* pneumonia, risk factors, severe *Mycoplasma pneumoniae* pneumonia

## Abstract

**Background:**

Bronchiolitis obliterans is a chronic obstructive lung disease characterized by irreversible airway damage, which leads to a poor prognosis. At present, there are relatively few studies on the risk factors for bronchiolitis obliterans caused by *Mycoplasma pneumoniae* in children. We investigated the clinical manifestations and risk factors for the development of bronchiolitis obliterans in children with severe *Mycoplasma pneumoniae* pneumonia with the aim of early intervention and improved prognosis.

**Methods:**

This study retrospectively analyzed 35 children with severe *Mycoplasma pneumoniae* who developed bronchiolitis obliterans hospitalized at the Children’s Hospital Affiliated with Zhengzhou University from January 2022 to November 2023 as the study subjects. In addition, 137 children with severe *Mycoplasma pneumoniae* did not develop bronchiolitis obliterans during the same period as the control group. We performed a multivariate logistic regression analysis of the clinical data of the two groups of patients to determine the independent risk factors for the development of bronchiolitis obliterans in children with pneumonia.

**Results:**

The age of the children in the bronchiolitis obliterans group was 52 (21, 83) months, the male to female ratio was 3:2, and the number of days of hospitalization was 11 (7, 15) days. The results of multivariate logistic regression analysis revealed that younger age (in months), the combination of viral infections, and the presence of the three-concave sign, pulmonary atelectasis, and bronchiectasis were independent risk factors for the development of bronchiolitis obliterans in children with severe *Mycoplasma pneumoniae*. The predictive value of the results of the multivariate logistic regression analysis for the area under the ROC curve assessment showed an AUC = 0.859 (95% CI, 0.788–0.931).

**Conclusion:**

The risk of bronchiolitis obliterans is greater in children with severe *Mycoplasma pneumoniae* at a younger age, with combined viral infections, and with manifestations such as the three-concave sign, pulmonary atelectasis, and bronchiectasis, and we should be vigilant for bronchiolitis obliterans so that early diagnosis can be made and timely intervention can be performed to treat the disease.

## Introduction

*Mycoplasma pneumoniae* (MP) is an important infectious agent of the lower respiratory tract that can occur at any age (1, 2). *Mycoplasma pneumoniae* pneumonia (MPP) is a common pathogen of community-acquired pneumonia in school-aged children and young adults (3). It accounts for approximately 20–40% of community-acquired pneumonia cases (4). Most children with MPP have mild clinical symptoms and are self-limiting, but a small percentage progress to severe *Mycoplasma pneumoniae* pneumonia (SMPP). The incidence of SMPP has increased in recent years (5–7). Owing to the severe progression of this disease, it has received much public attention. SMPP is usually accompanied by various serious complications and sequelae, such as pulmonary embolism, necrotizing pneumonia, and bronchiolitis obliterans (BO).

BO is a chronic obstructive lung disease in which the lumen is narrowed and occluded due to inflammation and peribronchiolar fibrosis (8). It is characterized by irreversible airway damage (8, 9). Children with BO often experience recurrent respiratory infections, as well as symptoms such as wheezing, shortness of breath, and dyspnea. Recurrent respiratory infections can also place a greater burden on the child’s family.

There are 3 main types of BO: postinfectious BO (PIBO), postlung transplant BO, and postbone marrow transplant or hematopoietic stem cell transplant BO (10). Among these, PIBO is the most common type of BO in children. The occurrence of PIBO is most often associated with adenovirus infection, followed by MP infection (11). However, few studies have been conducted to analyze the clinical characteristics and risk factors for the development of BO in children with SMPP (12). Therefore, we summarize the clinical characteristics of children with SMPP who develop BO sequelae and explore the risk factors for the development of BO to provide clinicians with some reference for early identification and intervention, thereby improving the prognosis and quality of life of these children.

## Materials and methods

### Ethics approval and consent to participate

This study was approved by the Ethics Committee of Henan Provincial Children’s Hospital (2025-KY-0037-001). Given that the study was retrospective, informed consent was not needed. The study adhered to the ethical standards of the Declaration of Helsinki.

### Study population

In this study, we collected data from children with SMPP (35 patients) who were hospitalized at the Affiliated Children’s Hospital of Zhengzhou University from January 2022 to November 2023 and who developed BO sequelae during the one-year follow-up. We used a random number table to randomly select children with SMPP who did not develop BO during the same period (137 cases) as the control group.

### Inclusion and exclusion criteria

The diagnostic criteria for MPP are as follows (13): (1) fever, cough, abnormal breath sounds, and dry and wet rales in the lungs; (2) typical chest imaging findings, such as interstitial infiltrates, segmental and lobar solid lesions, and hilar lymph node enlargement; and (3) positive tests for MP-DNA or RNA.

**Figure 1:**
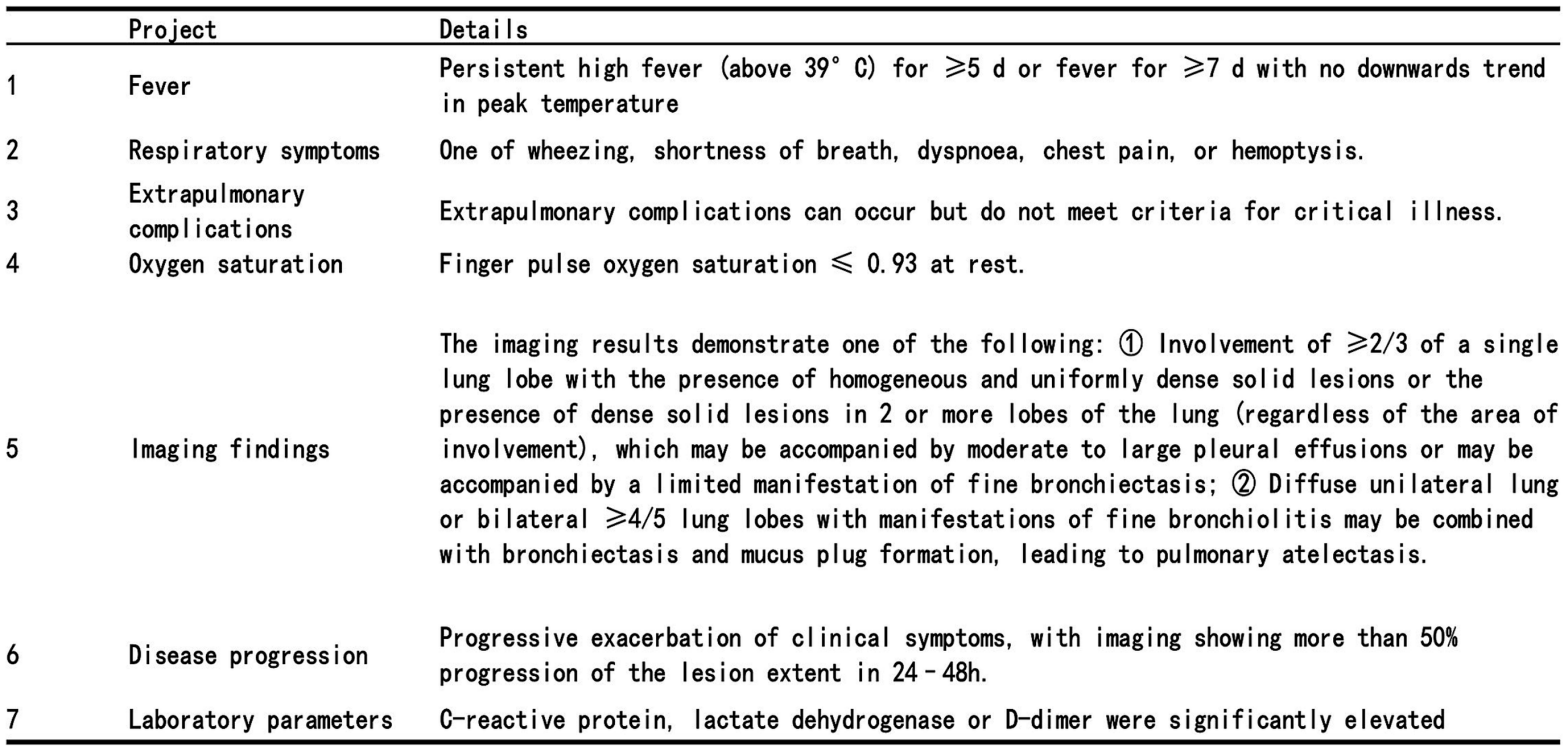
SMPP diagnostic criteria. A diagnosis is made when any one of the above criteria is satisfied.

The diagnostic criteria of SMPP are as follows (13, 14): See Figure 1 for specific diagnostic criteria.

The diagnostic criteria for PIBO are as follows (8): (1) Antecedent history: previous history of pneumonia. (2) Presence of persistent or recurrent wheezing or coughing, shortness of breath, dyspnoea, and exercise intolerance. Widespread wheezing and wet rales are audible in both lungs and persist for more than 6 weeks, with a poor response to bronchodilators. (3) Chest CT shows typical mosaic perfusion signs, bronchiectasis, and bronchial wall thickening. Pulmonary function shows small airway obstructive ventilatory dysfunction or mixed ventilatory dysfunction, and bronchodilator tests are mostly negative.

The inclusion criteria for the BO group were as follows: age greater than 1 month and less than 18 years; and meeting the diagnostic criteria for BO and the first diagnosis of BO at our hospital.

The inclusion criteria for the control group were as follows: age greater than 1 month and less than 18 years and no BO at 1 year of follow-up.

The exclusion criteria were as follows: (1) other serious diseases, such as connective tissue diseases, malignant tumors, hematologic diseases, or a history of bone marrow transplantation or organ transplantation; (2) underlying lung diseases, such as congenital malformation of bronchopulmonary development, bronchial asthma, or tuberculosis; and (3) incomplete clinical information or loss to follow-up.

### Data collection

We retrospectively collected clinical data from 172 children with SMPP in the acute phase of pneumonia during hospitalization: (1) general information, such as sex, age in months, and days of hospitalization; (2) clinical manifestations, such as duration of fever, fever peaks, cough, wheezing, shortness of breath, three-concave signs, dyspnoea, and other manifestations; (3) chest CT and fiberoptic bronchoscopy; (4) laboratory tests, such as mixed infections, blood counts, coagulation, biochemical indicators, immune function, and inflammatory indicators; and (5) therapeutic measures, including oxygen and mechanical ventilation.

### Statistical analyses

We used SPSS software (version 21.0) for the statistical analysis. Quantitative information that conformed to a normal distribution was expressed as the mean ± standard deviation, and comparisons of differences between groups were performed via independent samples t tests. Quantitative information that did not conform to a normal distribution was expressed as the median P50 (P25, P75) and compared via the Mann–Whitney U test. Qualitative information is expressed as a percentage (%). Univariate logistic regression analysis was performed for statistically significant differences between the two groups. Then, we used multivariate logistic regression analysis to identify independent risk factors for the development of BO in children with SMPP. Moreover, we assessed the predictive value of the results by means of subject operating characteristic (ROC) curves. A bilateral *p* value <0.05 indicated a statistically significant difference.

## Results

### Comparison of the clinical data between the two groups of children

The age of the children in the BO group was 52 (21, 83) months, the male to female ratio was 3:2, and the number of days of hospitalization was 11 (7, 15) days. In the control group, the age of the children was 79 (46, 96) months, the male to female ratio was 1.28:1, and the number of days of hospitalization was 11.0 (9.0, 14.5) days. The age of the children in the BO group was significantly younger than that of those in the control group, and the difference was statistically significant. There was no statistically significant difference between the two groups of children in terms of duration of fever, fever peak, or cough. The percentage of children with wheezing, shortness of breath, dyspnoea, and three-concave signs was significantly greater in the BO group than in the control group, and the difference was statistically significant. The percentage of children with combined viral and bacterial infections was significantly greater in the BO group than in the control group, and the difference was statistically significant. In addition, adenovirus infection accounted for the largest proportion of combined viral infections in this study, accounting for 57.89%. At the same time, the proportion of children in the BO group with pulmonary atelectasis and bronchiectasis on chest CT was significantly greater than that in the control group, and the difference was statistically significant. The remaining chest CT manifestations and bronchoscopy results were not significantly different between the two groups of children. The differences in the levels of white blood cells, neutrophils, lymphocyte counts, C-reactive protein, D-dimer, and lactate dehydrogenase between the two groups of children were not statistically significant. The difference between the two groups of children in terms of the use of oxygen and mechanical ventilation was also not statistically significant. (Tables 1, 2).

**Table 1:** Comparison between the two groups of children in terms of baseline characteristics, clinical manifestations, and coinfections.

Factors	BO group (*n* = 35)	control group (*n* = 137)	*p*
Sex (Male)	21, 60.00%	77, 56.20%	0.686
Age in months	52 (21, 83)	79 (46, 96)	0.002
Days of hospitalization	11.00 (7.00, 15.00)	11.00 (9.00, 14.50)	0.607
Duration of fever (day)	10 (7, 12)	9 (6, 12)	0.859
Fever peaks (°C)	39.50 (39.00, 40.00)	39.55 (39.00, 40.00)	0.483
Cough	35, 100.00%	135, 98.50%	1.000
Wheezing	15, 42.90%	23, 16.80%	0.001
Shortness of breath	27, 77.10%	68, 49.60%	0.003
Dyspnoea	28, 80.00%	69, 50.40%	0.002
Three-concave sign	18, 51.40%	23, 16.80%	<0.001
Virus	19, 54.30%	31, 22.60%	<0.001
Adenovirus	11, 31.40%	13, 9.50%	0.002
Bacteria	9, 25.70%	14, 10.20%	0.034
Fungi	2, 5.70%	2, 1.50%	0.389

**Table 2:** Comparison of chest CT, bronchoscopy, laboratory tests and therapeutic aspects between the two groups of children.

Factors	BO group (*n* = 35)	Control group (*n* = 137)	*p*-values
Pulmonary consolidation	27, 77.10%	102, 74.50%	0.743
Pulmonary necrosis	5, 14.30%	27, 19.70%	0.462
Pulmonary atelectasis	6, 17.10%	7, 5.10%	0.041
Pulmonary embolism	2, 5.70%	10, 7.30%	1.000
Bronchiectasis	7, 20.00%	2, 1.50%	<0.001
Bronchial stenosis	7, 20.00%	18, 13.10%	0.304
Thickening of the walls of the bronchi	2, 5.70%	2, 1.50%	0.389
Pleural effusion	16, 45.70%	59, 43.10%	0.778
Pleural thickening	10, 28.60%	33, 24.10%	0.585
Number of bronchoscopies	1.00 (1.00, 2.00)	1.00 (0.00, 2.00)	0.509
Endobronchitis	28, 80.00%	96, 70.10%	0.243
Bronchial necrosis	3, 8.60%	24, 17.50%	0.194
Bronchial sputum suppository	8, 22.90%	36, 26.30%	0.679
WBC 10^9/L	9.90 (7.14, 13.64)	10.41 (7.53, 13.68)	0.601
N 10^9/L	5.60 (3.40, 9.23)	7.06 (4.38, 9.72)	0.175
LYC 10^9/L	2.02 (1.04, 5.10)	2.47 (1.38, 3.97)	0.968
C-reactive protein mg/L	2.99 (0.50, 18.13)	9.54 (1.05, 35.37)	0.063
D-dimer ug/ml	0.74 (0.43, 1.75)	0.82 (0.43, 2.18)	0.458
Lactate dehydrogenase U/L	412.00 (263.50, 604.28)	367.50 (304.50, 486.50)	0.688
Administration of oxygen	18, 51.40%	72, 52.60%	0.905
Mechanical ventilation	5, 14.30%	8, 5.80%	0.184

WBC, White blood cell count; N, Neutrophil count; LYC, Lymphocyte count.

### Risk factors and ROC curves for the development of BO in children with SMPP

**Figure 2:**
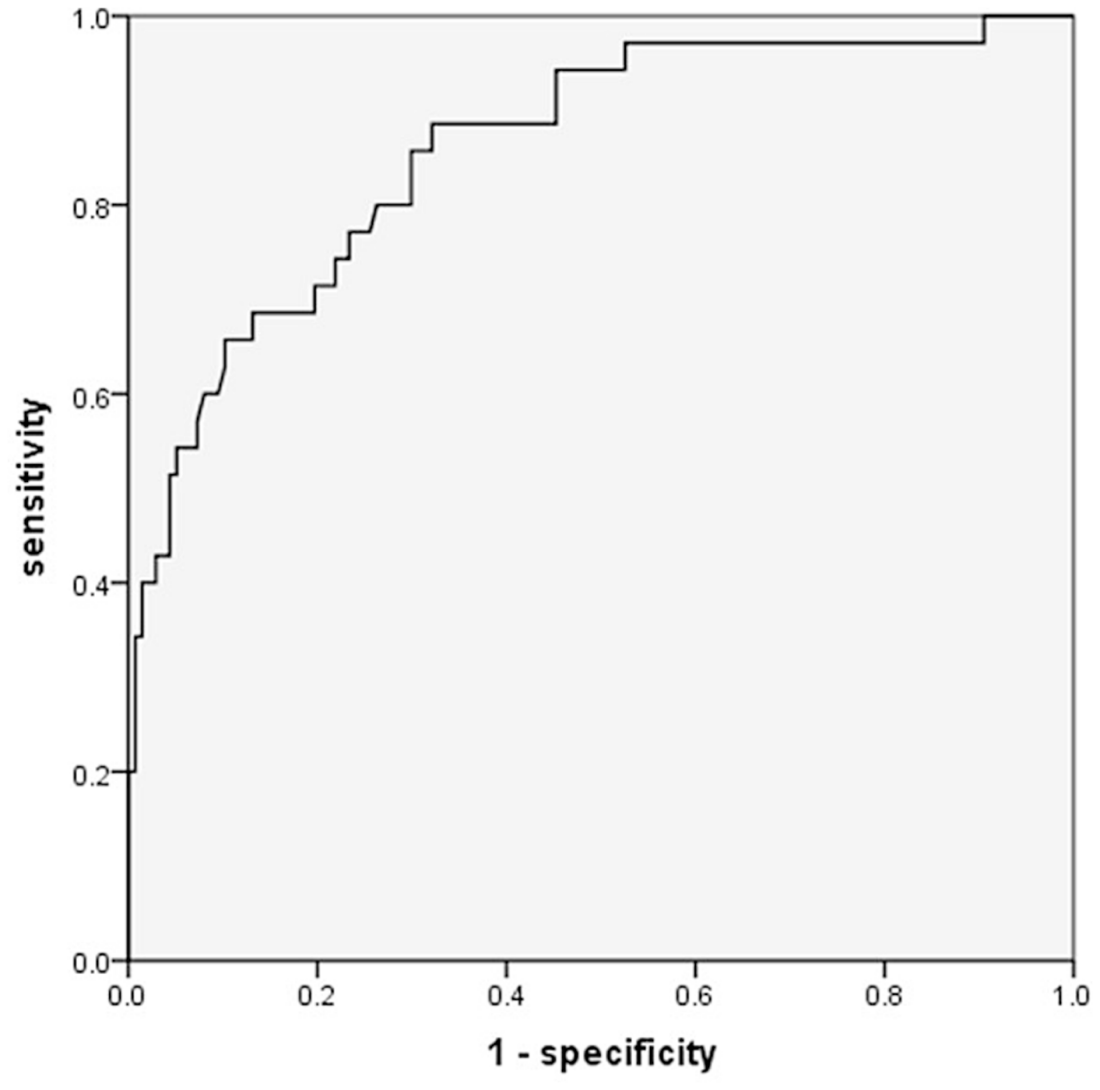
ROC curve for a multivariate logistic regression model. AUC = 0.859, 95% CI: 0.788–0.931, *p* < 0.001.

Using univariate logistic regression analysis, we found a statistically significant effect of nine risk factors on the occurrence of BO in children with SMPP. Younger age, wheezing, shortness of breath, dyspnoea, three-concave signs, a combination of viral and bacterial infections, and the development of pulmonary atelectasis and bronchiectasis are risk factors for the development of BO in children with SMPP. The results of multivariate logistic regression analysis revealed that younger age, combined viral infection, the presence of the three concave signs, pulmonary atelectasis, and bronchiectasis were independent risk factors for the development of BO in children with SMPP (Table 3). At the same time, a multicollinearity test was performed on the predictive model. The results showed that the variance inflation factors (VIFs) for age in months, combined viral infection, three concave signs, pulmonary atelectasis, and bronchiectasis were 1.175, 1.025, 1.151, 1.064, and 1.058, respectively. This suggests that multicollinearity in the model is mild. The AUC of the results of the multivariate logistic regression analysis was 0.859 (95% CI, 0.788–0.931), indicating good predictive value (Figure 2; Table 4).

**Table 3:** Risk factors for the development of BO in children with SMPP.

Factors	Univariate analysis		Multivariate analysis	
	*p*-values	OR (95% CI)	*p*-values	OR (95% CI)
Age in months	0.002	0.983 (0.972, 0.994)	0.034	0.985 (0.971, 0.999)
Wheezing	0.001	3.717 (1.661, 8.319)	/	/
Shortness of breath	0.005	3.425 (1.453, 8.070)	/	/
Dyspnoea	0.003	3.942 (1.613, 9.632)	/	/
Three-concave sign	<0.001	5.248 (2.358, 11.681)	0.001	5.193 (1.909, 14.128)
Virus	<0.001	4.060 (1.869, 8.824)	<0.001	5.781 (2.190, 15.261)
Bacteria	0.020	3.041 (1.190, 7.771)	/	/
Pulmonary atelectasis	0.023	3.842 (1.202, 12.286)	0.021	6.092 (1.315, 28.209)
Bronchiectasis	0.001	16.875 (3.328, 85.554)	<0.001	39.849 (5.242, 302.924)

**Table 4:** Predictive value of age in months, three-concave sign, viral infections, pulmonary atelectasis and bronchiectasis for combined BO in children with SMPP.

Factors	AUC	*p*-values	Cutoff	Sensitivity (%)	Specificity (%)	Youden index	95%CI
Age in months	0.332	0.002	59.50	0.400	0.299	−0.301	0.236, 0.427
Three-concave sign	0.673	0.002	/	0.514	0.832	0.346	0.565, 0.781
Virus	0.658	0.004	/	0.543	0.774	0.317	0.552, 0.765
Pulmonary atelectasis	0.560	0.273	/	0.171	0.949	0.120	0.448, 0.673
Bronchiectasis	0.593	0.091	/	0.200	0.985	0.185	0.478, 0.707
Combined indicators	0.859	<0.001	/	0.886	0.679	0.565	0.788, 0.931

## Discussion

PIBO is a chronic airflow obstruction syndrome resulting from damage to the lower respiratory tract caused by pathogen infection. Adenoviruses are the most common pathogens of PIBO. Currently, relevant studies have identified MP as the second most important pathogen of PIBO (12, 15). In studies conducted in Malaysia and Korea, 20% of PIBO cases were caused by MP infection (15). In recent years, the number of pediatric cases of MP infection leading to the development of BO has increased (16). In the study by Wang et al., the proportion of children with BO caused by MP infection was the same as that of children with BO caused by adenovirus infection, accounting for 37.5% of the children in this study (*n* = 16) (17). An increase in BO patients, with MP as a causative factor, may be associated with an increased incidence of MPP (12, 17). Therefore, for children with SMPP, we should identify the risk factors for BO at an early stage and make timely diagnoses and interventions to reduce the occurrence of PIBO and improve the prognosis (18). In this study, we derived five independent risk factors for the development of BO in children with SMPP via multivariate logistic regression analysis in the hope of providing experience in the early identification of PIBO.

The results of this study revealed that age in months was an independent risk factor for the development of BO in children with SMPP. The younger the child is, the greater the risk of BO. In the current study, it is generally recognized that PIBO occurs mostly in infancy (12). In the study by Liu et al., children who developed PIBO were younger than those in the control group (19). This finding is consistent with the present study. We hypothesize that the immune system is not yet fully developed in young children. Therefore, these patients are more susceptible to infection, and the airway damage caused by infection is more severe. Severe airway damage can further lead to BO (20). Therefore, in children with SMPP at a young age in months, one should be vigilant in the development of BO.

The three-concave sign is an independent risk factor for the development of BO in children with SMPP. In the present study, the percentage of children in the BO group who presented with the three-concave sign was significantly greater than that in the control group. This result suggests that children with SMPP who have the triple concave sign are more likely to develop BO. Several previous studies have shown that hypoxemia is a risk factor for PIBO (16, 19). The development of hypoxemia is associated with severe lung injury. If hypoxia is not corrected in children with the three-concave sign, it can further exacerbate airway inflammation and lung injury, which in turn promotes fibroblast overgrowth, aberrant tissue repair and remodeling of the fine bronchial walls, leading to BO (10, 16, 21, 22).

Mixed viral infections were found to be an independent risk factor for the development of BO in children with SMPP in our study. Additionally, in this study, adenovirus infection was the most common type of combined viral infection, accounting for 57.89% of all infections. Although adenovirus infection is a known risk factor for children with PIBO (23), few studies have reported on whether coinfection with adenovirus is a risk factor for the development of BO in children with MPP (24). A study by Lee et al. revealed an association between an increased risk of PIBO and coinfection with adenovirus in children with MPP (24, 25). It has also been shown that children with MPP and adenoviral infection are more likely to develop severe damage, such as airway mucosal erosion and exfoliation (24). In addition, we also found a significantly greater proportion of children with coinfections of bacteria in the BO group. Previous studies have shown that mixed bacterial infections are a risk factor for PIBO in children with pneumonia (19). As a result, children with mixed infections are sicker, have a longer duration of illness, have more severe airway damage and are more likely to have BO (26, 27).

In our study, pulmonary atelectasis was an independent risk factor for the development of BO in children with SMPP, and the risk of BO was greater in children with SMPP who had manifestations of pulmonary atelectasis. Pulmonary atelectasis is an intrapulmonary complication in children with MPP. On the one hand, it further leads to poor ventilation and localized hypoxia of the lung tissue (28, 29). In a hypoxic environment, the repair function of lung tissue is altered, resulting in an abnormal fibrotic response and remodeling of the fine bronchial walls, which in turn promotes the development of BO (21). On the other hand, pulmonary atelectasis exacerbates the airway inflammatory response, promotes fibrosis and occlusion of the bronchi, and triggers BO (21). Bronchiectasis was also an independent risk factor for the development of BO in children with SMPP in this study. Worsening bronchiectasis has been shown in previous studies to result in increased airway and systemic inflammation and is associated with progressive lung injury (30). We hypothesize that this process leads to airway remodeling, which in turn promotes BO.

In addition, in our present study, wheezing, shortness of breath, and dyspnea occurred in a significantly greater percentage of children in the BO group than in the control group. Univariate logistic regression analysis revealed that all the variables were statistically significant for the occurrence of BO sequelae in children with SMPP. In a study by Zhao et al., it was shown that children with MP infections need to be considered for BO if clinical signs such as wheezing and dyspnea do not subside within the expected time or if pulmonary function does not improve (22). Several previous studies have shown that young age, wheezing, shortness of breath, hypoxemia, high lactate dehydrogenase levels, and mechanical ventilation are risk factors for the development of BO in children with pneumonia (19, 20, 31). Among them, wheezing, shortness of breath, hypoxemia, and mechanical ventilation were shown to be the highest risk factors for PIBO in a study by Liu et al. (19). The histopathological basis of wheezing in children with pneumonia is inflammation-induced airway narrowing, and inflammation leads to airway epithelial swelling, congestion, hypersecretion, and scarring (20). Damage to and repair of the bronchiole may lead to narrowing and occlusion of the lumen. We therefore hypothesized that persistent wheezing may promote the development of BO. Moreover, hypoxia symptoms such as shortness of breath and dyspnea further aggravate damage to the airway epithelium, leading to dysregulation of epithelial repair, overactivation of fibroblasts, stimulation of fibrous proliferation, and tissue remodeling, which promote the development of BO (32).

This study has several limitations. In this study, owing to the rarity of children with PIBO, the study had a small sample size and included children from a single center. Larger samples and multicenter studies are needed to further validate the results. At the same time, no comparative analysis was conducted regarding treatment approaches, such as whether to administer high-dose steroid pulse therapy or use gamma globulin; further research is needed in the future.

In conclusion, infants with SMPP who are small for their age in months, presenting with the three-concave sign, coinfection with viral infections, the presence of pulmonary atelectasis, and bronchiectasis are at higher risk of developing PIBO. We should be highly vigilant for the occurrence of PIBO to achieve early recognition and intervention to avoid irreversible airway obstruction, thus improving the quality of life of these children.

## Data Availability

The raw data supporting the conclusions of this article will be made available by the authors, without undue reservation.
